# Ibuprofen-Loaded Silver Nanoparticle-Doped PVA Gels: Green Synthesis, In Vitro Cytotoxicity, and Antibacterial Analyses

**DOI:** 10.3390/gels10020143

**Published:** 2024-02-14

**Authors:** Ezgi Altınay, Fatma Zehra Köse, Sezen Canım Ateş, Kadriye Kızılbey

**Affiliations:** 1 Institute of Science and Engineering, İstanbul Yeni Yüzyıl University, İstanbul 34010, Türkiye; 2 Biomedical Engineering Department, Faculty of Engineering and Architecture, İstanbul Yeni Yüzyıl University, İstanbul 34010, Türkiye; 3 Department of Natural Sciences, Faculty of Engineering and Natural Sciences, Acıbadem University, İstanbul 34752, Türkiye

**Keywords:** drug release, polyvinyl alcohol, hydrogel, kinetic models

## Abstract

In contrast to conventional drug delivery systems, controlled drug release systems employ distinct methodologies. These systems facilitate the release of active substances in predetermined quantities and for specified durations. Polymer hydrogels have gained prominence in controlled drug delivery because of their unique swelling–shrinkage behavior and ability to regulate drug release. In this investigation, films with a hydrogel structure were crafted using polyvinyl alcohol, a biocompatible polymer, and silver nanoparticles. Following characterization, ibuprofen was loaded into the hydrogels to evaluate their drug release capacity. The particle sizes of silver nanoparticles synthesized using a green approach were determined. This study comprehensively examined the structural properties, morphological features, mechanical strength, and cumulative release patterns of the prepared films. In vitro cytotoxicity analysis was employed to assess the cell viability of drug-loaded hydrogel films, and their antibacterial effects were examined. The results indicated that hydrogel films containing 5% and 10% polyvinyl alcohol released 89% and 97% of the loaded drug, respectively, by day 14. The release kinetics fits the Korsmeyer–Peppas model. This study, which describes nanoparticle-enhanced polyvinyl alcohol hydrogel systems prepared through a cost-effective and environmentally friendly approach, is anticipated to contribute to the existing literature and serve as a foundational study for future research.

## 1. Introduction

The effective dosage range of drugs in the body varies, and over- or underdosing can lead to undesirable side effects. Unlike conventional methods, the precise control of drug release enables the maintenance of the drug concentration within the therapeutic range, enhancing treatment efficacy and promoting cost-effective drug usage [1]. In addition to achieving targeted and selective drug delivery, there is growing demand for long-term release strategies. The evolving field of biomedicine has witnessed the development of drug carrier systems aimed at mitigating these challenges [2]. Among the most effective solutions for these objectives are controlled drug release systems. These systems are designed to ensure the presence of the desired drug in a specific area of the body, in the required quantity, and for the designated duration.

Controlled drug release systems are designed to minimize the active drug ingredient, prolong its residence time within the living system, and mitigate potential harmful side effects associated with varying drug concentrations in patients. Among various materials, polymers are extensively employed in these systems because of their inherent similarities to natural biological systems [3]. Achieving controlled drug release often involves loading active ingredients into polymers using various methods, which necessitates careful polymer selection, with biocompatibility being a crucial factor [4]. When selecting polymers for drug-carrying systems, consideration must be given to their biological properties as well as their mechanical and physical characteristics. Key physical and mechanical properties include swelling behavior, tensile and compression strength, geometric structure, elasticity, and rupture and tear resistance [5]. Polyvinyl alcohol (PVA) is a synthetic polymer with desirable attributes for controlled drug release systems. Being biocompatible, water-soluble, biodegradable, odorless, and nontoxic, PVA possesses high tensile strength and flexibility, making it easy to process. Its crystalline structure, coupled with the ability to crosslink with hydroxyl groups, contributes to its essential physical properties, including elasticity, high swelling in aqueous solutions, adhesiveness, and excellent film-forming capacity [6]. PVA can undergo crosslinking, but the process is typically enhanced or facilitated by the use of a crosslinking agent. Crosslinking describes the formation of chemical bonds between polymer chains, which often improves the mechanical and thermal properties of polymers. In the case of PVA, common crosslinking agents include borates, aldehydes (such as formaldehyde or glutaraldehyde), and epoxides. These agents react with the hydroxyl groups in the PVA polymer chain to create crosslinks. PVA can undergo some degree of self-crosslinking through the formation of intramolecular hydrogen bonds between hydroxyl groups along the polymer chain [7,8]. However, the choice between self-crosslinking and external crosslinking depends on the desired material properties and the specific requirements of the intended application. Notably, PVA can be converted into a hydrogel, offering flexibility, softness, minimal interaction with blood cells and proteins, and low surface tension [9]. Hydrogels, which are used in diverse applications, such as wound dressings, contact lenses, controlled drug release systems, and artificial blood vessels, can undergo structural changes, exhibiting swelling and shrinkage properties depending on various conditions [10]. However, hydrogels often lack sufficient barrier properties against bacteria, necessitating the incorporation of antimicrobial functionality. Various techniques, including the addition of nanoparticles, have been employed to confer antimicrobial properties to hydrogels [11]. In the quest for antimicrobial hydrogels, silver nanoparticles (AgNPs) play a prominent role. AgNPs can be synthesized using biological, chemical, or physical methods. Biological synthesis, particularly green synthesis using nontoxic molecules such as plant extracts, has emerged as a practical and environmentally friendly approach. This method enables the production of nanoparticles without the need for high pressure, temperature, energy, or toxic chemicals, making it a cost-effective alternative to many physical and chemical methods [12,13]. 

In this study, AgNPs were derived from the plant *Equisetum telmateia* via green synthesis. *E. telmateia*, commonly known as horsetail, belongs to the Equisetaceae family, and it is renowned for its traditional use in treating urinary system diseases, stomach pain, eczema, and mouth infections [14]. The plant is rich in biologically active compounds, and the existing literature highlights its antioxidant and antimicrobial effects. Notably, studies by Stajner et al. underscored the antimicrobial and potent antioxidant properties of polar extracts from *E. telmateia*, suggesting its potential as a valuable source of antioxidants with robust cleansing abilities [15,16].

Delving into the extraction yield, chemical composition, and therapeutic potential of *E. telmateia*, this research sought to unlock the mysteries of its bioactive compounds. The supercritical fluid extract displayed exceptional potency, including low minimum inhibitory and bactericidal concentrations, and it inhibited the growth of all bacteria and fungi tested. This significantly contributes to the plant’s potential therapeutic applications. Traditionally employed for various health issues in Asia, northwest Africa, and North America, the plant’s abundant phenolic compounds position it as a promising source of diverse health substances [15]. This investigation aimed to clarify the active substance content and biological effects of *E. telmateia*. Phenolic compounds, particularly flavonoids derived from plants, are essential natural compounds recognized for their antioxidant and antimicrobial properties, and they have applications in medicine, pharmacy, and the food industry. This study addressed a gap in the existing literature, as limited data are available on the biological activity of *E. telmateia*. Specifically, this research examined the total soluble phenolic content and flavonoid concentrations in *E. telmateia* extracts using spectrophotometric methods. The results revealed that the total phenolic content, assessed with Folin–Ciocalteu reagent, ranged from 129.0 to 262.7 mg gallic acid/g. The flavonoid concentrations in various extracts varied from 112.6 to 199.8 mg rutin/g. Notably, this study marks the first exploration of the antimicrobial activities of the acetone, ethyl acetate, and methanol extracts of *E. telmateia*. These findings highlight the significant antimicrobial activity of *E. telmateia*, which is particularly noteworthy for its potential as a source of antimicrobial substances. The methanol extract displayed superior efficiency against bacteria, especially Gram-positive strains. Overall, all extracts exhibited substantial antibacterial activity against Gram-positive bacteria and weak-to-moderate activity against other microorganisms [17]. The scientific realm is teeming with instances of the efficacy of plant extracts in the synthesis of AgNPs across diverse botanical families, including Acanthaceae, Amaranthaceae, Apocynaceae, Asphodelaceae, Asteraceae, Burseraceae, Dioscoreaceae, Euphorbiaceae, Fabaceae, Lamiaceae, Moraceae, Myrtaceae, Poaceae, Rutaceae, and Solanaceae. Notably, *Equisetum arvense* has been extensively studied for its biological activities, whereas other members of the *Equisetum* genus have been traditionally used in various medicinal applications. A study by Batir-Marin et al. is the sole study in the literature focusing on metal nanoparticle synthesis using species from the *Equisetum* genus, including *E. telmateia*, through the green synthesis method. Motivated by the myriad applications of AgNPs and the unique advantages offered by *Equisetum* plants in their synthesis, the present research explored the potential of select horsetail species in AgNP synthesis. This investigation aimed to elucidate the applications of horsetail in medicine, capitalizing on its potential antioxidant and antitumor activities, as well as environmental protection through photocatalytic capabilities. Consequently, this study performed a structural and morphological characterization of AgNPs derived from the ethanolic extracts of three underexplored *Equisetum* species: *E. pratense* Ehrh., *E. sylvaticum* L., and *E. telmateia* Ehrh. Significantly, this research marks the first report on AgNPs obtained from any of these three *Equisetum* species, underscoring the novelty of the study [18]. Ethanol extraction was performed in this study. Unlike the study by Batir-Marin et al. [18], green synthesis was performed in our study using aqueous extraction of the plant instead of ethanol extraction. In this manner, our work differs from prior studies.

Controlled release systems can be prepared using various methods depending on the specific drug. When selecting the preparation method, the structure and properties of the active ingredient must be carefully considered. Ibuprofen, a nonsteroidal anti-inflammatory drug and analgesic, was chosen for this study. With a molecular weight of 206.28 g/mol and a closed formula of C_13_H_18_O_2_, the efficacy and safety of ibuprofen have been demonstrated since its introduction. Its high patient compliance, applicability across age groups, effectiveness at low doses, and relatively short half-life in the body (1.6–2.5 h) make it suitable for controlled drug release applications [19,20,21]. Although ibuprofen is generally considered safe, its usage has been linked to severe adverse effects, particularly with increasing doses. The need to reduce the dosage of ibuprofen and extend its in vivo activity are recognized as a critical area of research. Prior studies discussed diverse approaches to address the challenges associated with ibuprofen usage, aiming to enhance its therapeutic efficacy and mitigate potential adverse effects. In a study by Chen et al., a novel ibuprofen–cellulose nanofibril drug delivery system was developed, and pH-responsive behavior for controlled drug release was detected over 24 h. The study revealed 90% cumulative in vitro drug release under alkaline conditions [22]. Similarly, Li et al. explored the modification of TiO_2_ nanotube arrays with polydopamine to improve ibuprofen loading and release properties. This approach provided promising results for sustained drug release, which is particularly advantageous in bone implant therapies [23]. Moreover, the study by Bensouiki et al. delved into the synthesis of nanoparticles based on sodium alginate and chitosan for ibuprofen encapsulation, revealing pH-sensitive behavior and controlled drug release [24]. In another study, Ávila et al. employed carbon nanocapsules as carriers for sodium ibuprofen release, highlighting their high drug loading capacity and rapid release (approximately 100% of initial sodium ibuprofen), making them potential candidates for oral drug administration [25]. Finally, de Freitas Lima and Pegorin conducted a study focusing on the development of ibuprofen-loaded membranes using natural rubber latex (NRL). The IBU–NRL membrane exhibited favorable characteristics, with no hindrance to drug release and suitable mechanical properties for cutaneous adhesive applications. Hemocompatibility was confirmed, and the release profile featured an initial burst effect followed by stable release over 96 h. Overall, the IBU–NRL membrane displayed significant potential as a novel adhesive for treating inflammatory processes and injuries [26]. Collectively, these studies contribute valuable insights into tailored drug delivery systems, emphasizing controlled release kinetics and enhanced therapeutic outcomes for ibuprofen.

The release profile of ibuprofen was investigated by developing a hydrogel film based on PVA doped with AgNPs. Characterization of the AgNPs used in the films was performed using a Zetasizer device and ultraviolet–visible (UV–Vis) and Fourier-transform infrared (FT-IR) spectrophotometers. PVA hydrogel films were prepared using the freeze–thaw technique. FT-IR spectra were employed for chemical and structural analysis of the prepared films, whereas scanning electron microscopy (SEM) was used to elucidate their morphological properties. Subsequently, swelling and tensile tests were performed to evaluate the mechanical strength of the films. The drug release profiles of the hydrogel films were examined, followed by assessments of their cytotoxicity and antibacterial effects.

## 2. Result and Discussions

**Figure 1:**
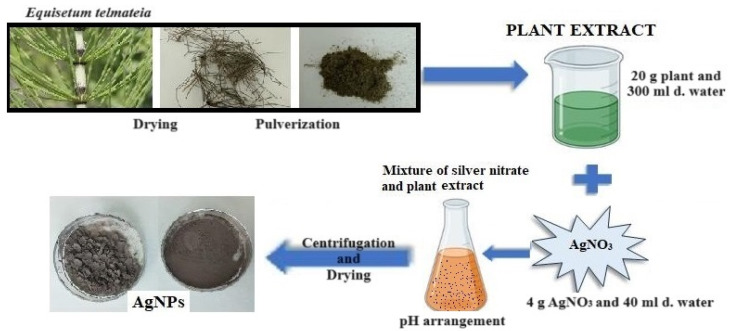
Schematic of green synthesis.

In this study, hydrogel-structured films were prepared using PVA polymer by doping AgNPs obtained via green synthesis from *E. telmateia* (Figure 1). Both AgNPs and PVA hydrogels prepared by the freeze–thaw technique were characterized using various methods. 

### 2.1. Characterization of AgNPs 

**Figure 2:**
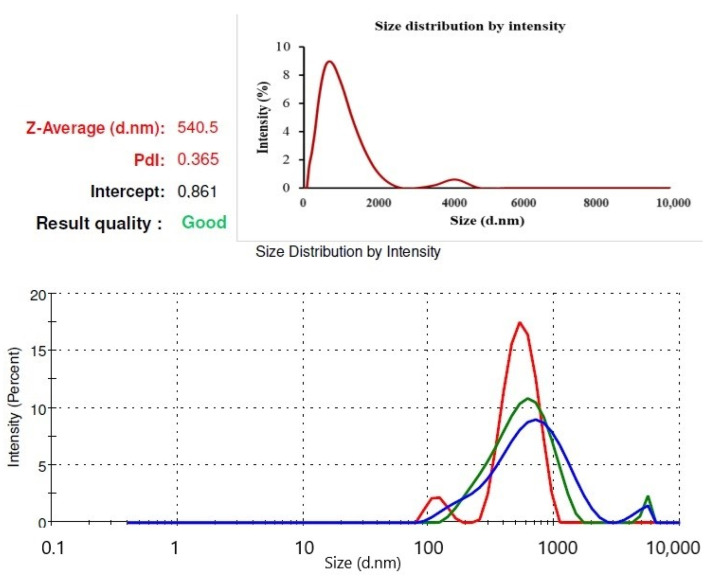
Size distribution of AgNPs by intensity.

A Zetasizer device, SEM, UV–Vis, and FT-IR spectrophotometers were used to characterize AgNPs. The size distribution of AgNPs by intensity is presented in Figure 2, and 3 measurements are taken by the device. These are specified in 3 different colors: red, green and blue. As a result of the measurements, the values are determined by calculating the average. The analysis was performed in three repetitions, and the results were presented as the average of the obtained values. The size of the nanoparticles was 540.5 ± 27.72 nm, and the polydispersity index was 0.365. Because the polydispersity index of the nanoparticles was less than the value of 0.7 stated in the literature, nanoparticles were used in the experiments [27]. 

**Figure 3:**
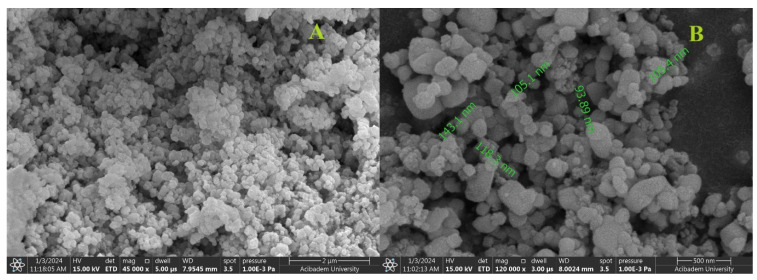
SEM images of AgNPs at 45,000× (**A**) and 120,000× (**B**) magnifications.

The SEM image in Figure 3 indicates the production of uniformly dispersed nanoparticles with a high density of AgNPs. Under low magnification (Figure 3A), a significant density of nanoparticles is discernible, confirming the synthesis of monodisperse nanoparticles within a size range of 100–200 nm. Clearer images of the AgNPs are presented at higher resolution and their sizes are indicated in the Figure 3B in green. The micrographs exhibit consistency in both the size and shape of the AgNPs, but in Zeta sizer analysis, the Z-average of AgNPs was 540.5 ± 27.72 nm, which is not fully consistent with these analyses. This is thought to be due to aggregation occurring during sample preparation and the fact that nanoparticles have a polydisperse distribution. Predominantly in SEM analysis, the nanoparticles exhibit a spherical shape; however, some also have elliptical configurations. This comprehensive analysis provides the characteristics of the AgNPs, emphasizing their uniformity and stability.

**Figure 4:**
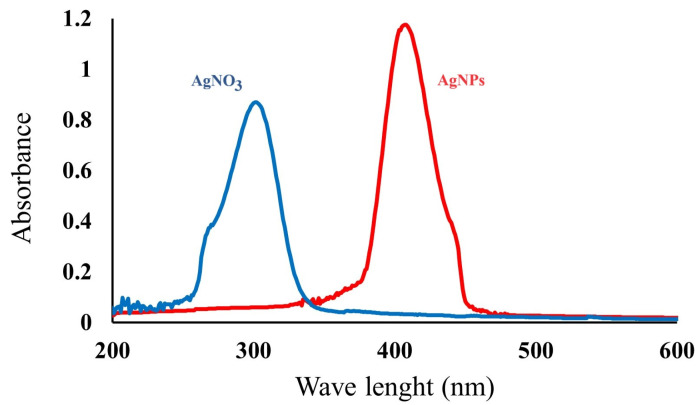
UV–Vis spectrum of AgNO_3_ and AgNPs.

The formation of AgNPs was traced using a UV–Vis spectrophotometer (Figure 4). The peak of AgNPs was at 375–450 nm, consistent with the literature [28]. The maximum peak point of the surface plasmon band was at around 410 nm. The diminutive and globular AgNPs exhibited absorption around 400 nm with well-defined peaks, whereas the more substantial AgNPs displayed a redshift (absorption at longer wavelengths) accompanied by broader peaks [29]. This phenomenon serves as an indication of the enduring stability of AgNPs, as the peaks gradually diminish in intensity and widen. Simultaneously, secondary peaks emerge at higher wavelengths, indicating particle aggregation. In Figure 4, the peak at 255–345 nm refers to AgNO_3_ solution. UV–Vis is not sufficient to give an overall picture of the synthesized AgNPs; however, the shift in the AgNO_3_ peak provides information about a change in the structure. 

**Figure 5:**
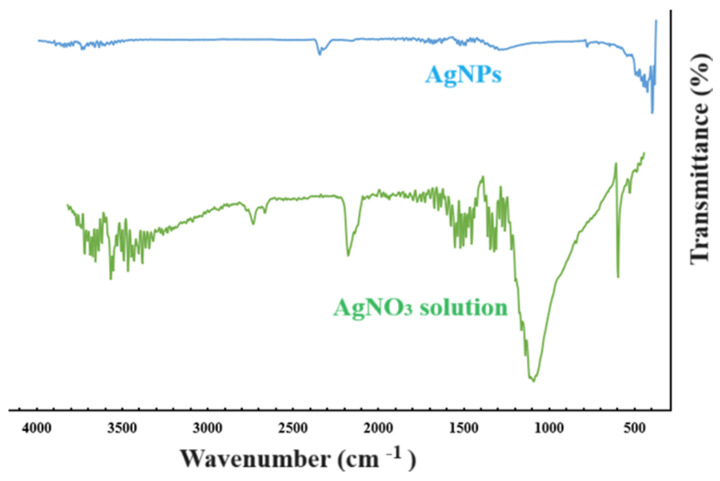
FT-IR spectra of AgNPs and AgNO_3_ solution.

AgNPs were synthesized using AgNO_3_ solution via the green synthesis method. For this reason, in FT-IR measurements, starting from AgNO_3_ solution, the chemical structures of AgNPs, AgNPs/PVA hydrogel films (B1 and E1), free ibuprofen, and ibuprofen-loaded AgNPs/PVA hydrogel films were analyzed. The comparative FT-IR spectra of AgNO_3_ solution and AgNPs are presented in Figure 5. The spectrum of AgNO_3_ solution featured various peaks at 3749.61, 2364.72, 1651.06, 1519.9, 1288.44, and 798.52 cm^−1^. However, with the formation of AgNPs after the reduction of silver, the distinct peaks in the AgNO_3_ spectrum became smaller, whereas new peaks formed at 667.37, 567.07, and 447.48 cm^−1^ after the band at 730 cm^−1^. Other peaks included a C=C stretching peak at 1680–1620 cm^−1^ and an aromatic C–H stretching peak at 2364.72 cm^−1^. The peak near 630.42 cm^−1^ was assigned to the C–C–H group of alkynes. It is believed that the peaks not present in AgNPs arose from the functional groups present in the plant extract structure [30].

Distinct peaks were seen for AgNPs at 420 and 405 cm^−1^. FT-IR spectroscopy is important for investigating the functional groups of compounds. In this study, it was used to characterize the plant extract and the nanoparticles synthesized from the extract. The results strongly support our hypothesis that various phytochemical components, such as alkaloids, amino acids, flavonoids, saponins, steroids, glycosides, carbohydrates, tannins, and phenolic compounds, are involved in the formation and stabilization of nanoparticles. A change in density and a small shift in the spectrum of the nanoparticles were observed. This was believed to be attributable to the coordination of phytochemicals on the metal surface [30]. 

### 2.2. Characterization of the PVA Hydrogel Films

#### 2.2.1. FT-IR Spectroscopy 

**Figure 6:**
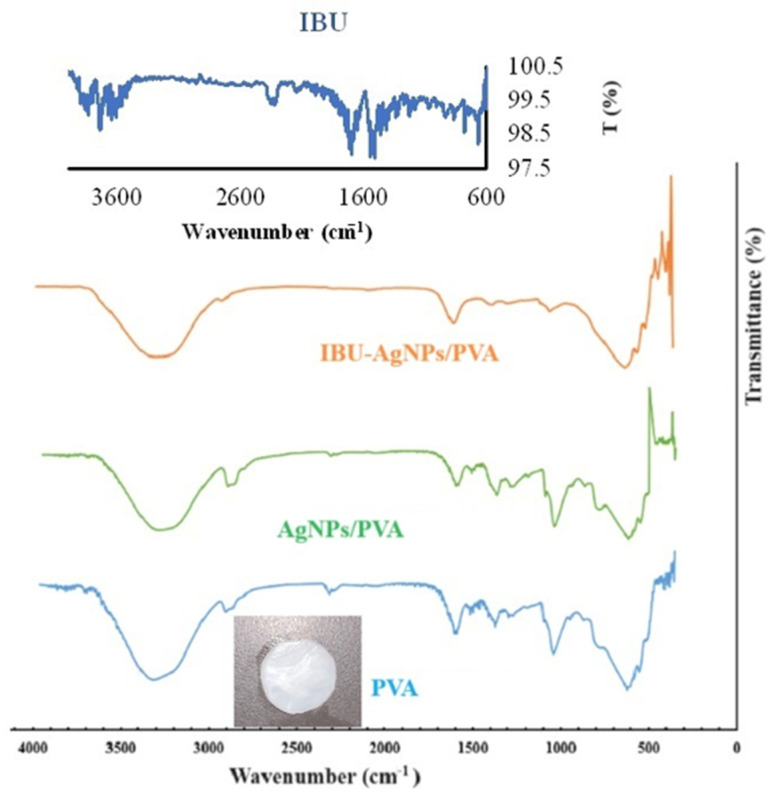
FT-IR spectra of PVA hydrogel films and ibuprofen (IBU).

The FT-IR spectra of the AgNP-doped PVA and PVA hydrogel films are presented in Figure 6. The characteristic absorption peaks for PVA are 3309 (O-H stretching), 2939 (asymmetric stretching of CH_2_), 2914 (symmetric stretching of CH_2_), 1647 (attributable to water absorption), 1417 (CH_2_ bending), 1325 (swaying by δ(OH), CH shaking), 1141 (shoulder stretching of C-O) (crystalline sequence of PVA), 1085 (stretching of C-O and bending of OH) (amorphous sequence of PVA), 914 (CH_2_ shaking), and 817 cm^−1^ (C-C stretching) [31,32,33].

In FT-IR analysis, the spectrum of ibuprofen featured distinct bands in the range of 1720–1500 cm^−1^. In particular, an intense and well-defined band specific to ibuprofen was observed at 1720 cm^−1^, and this band originated from the carbonyl stretching of the oxopropanoic acid group [34]. The acquired spectrum featured sharp characteristic peaks at 1720 cm^−1^, corresponding to the carboxyl acid found in ibuprofen. Other smaller peaks in the region of 1200–1000 cm^−1^ are indicative of a benzene ring. Analysis of the spectrum of ibuprofen revealed the presence of significant bands. A characteristic broad band of acid O–H stretching was observed in the 3200–2500 cm^−1^ region. O–H bending of the same group was observed at 935.82 cm^−1^. Carbonyl stretching vibration (C=O) was observed at 1720.63 cm^−1^, and C–O stretching vibration was observed at 1230.88 cm^−1^ [35]. 

The FT-IR spectrum of the IBU-AgNP/PVA film is also presented in Figure 6. When the graphical results of the PVA and AgNP/PVA films obtained from the FT-IR spectra were evaluated, the hydrogel films were found to not undergo any change in their copolymeric properties. When comparing the FT-IR spectra of films with and without the active ingredient, no major peak change was observed between the graphs. When examining films containing ibuprofen, the film was found to maintain its chemical properties. When the graphical results of PVA, AgNP/PVA, and IBU-AgNP/PVA films were evaluated, it was concluded that AgNP and ibuprofen were not collected on the surface of the polymer, and they were instead trapped inside the polymer. The sharp, characteristic peaks of ibuprofen were not present in its current spectrum after being loaded into the film. In addition, when the obtained spectra were examined, it was thought that ibuprofen did not interact with the polymer and metal nanoparticles, as no peak formation indicating the existence of new interactions was observed. 

#### 2.2.2. Swelling Test 

**Figure 7:**
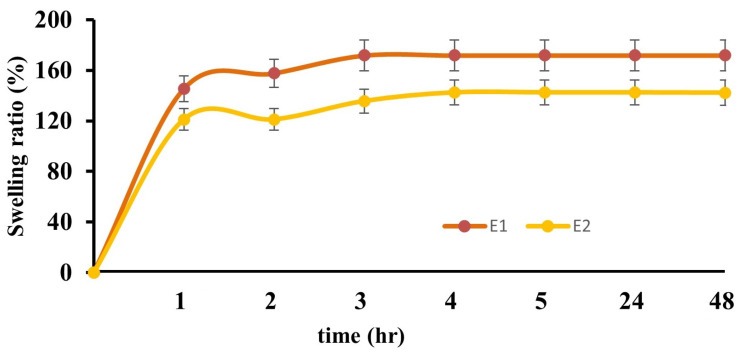
Graph of the swelling ratios of E1 and E2 hydrogel films.

**Figure 8:**
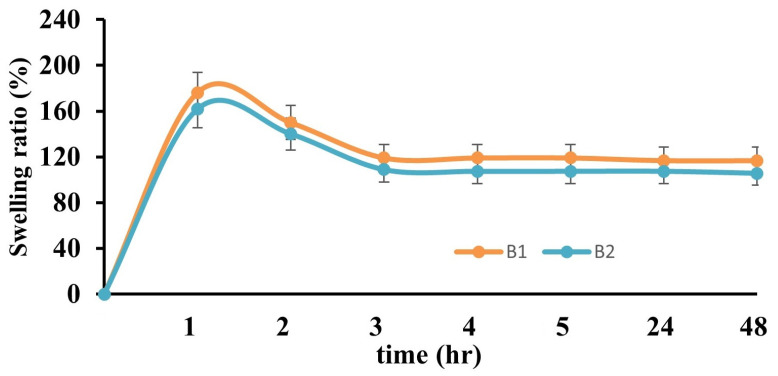
Graph of the swelling ratios of B1 and B2 hydrogels.

The swelling test results of 5% (*w/v*) and 10% PVA films (B2 and E2) and their films doped with AgNPs (B1 and E1) are presented in Figure 7 and Figure 8, respectively. The swelling behavior of the hydrogels changed with changes in the PVA ratios of the films and the addition of AgNPs. When the maximum swelling percentages of B1, B2, E1, and E2 films were compared, it was found that the degree of swelling decreased as the PVA ratio increased. This result is compatible with the literature. This is related to the increase in the number of PVA–PVA bonds as the amount of PVA increases. Although the maximum swelling percentage of the hydrogel (B2) containing 5% (*w/v*) PVA was high, the degree of swelling decreased with increasing time. However, the maximum level was reached in the first hour, followed by a decrease and an equilibrium swelling state in the fourth hour. This is because PVA is water-soluble, and its degree of swelling increases because of free hydroxyl groups. It was determined that the swelling degree of the hydrogel (E2) containing 10% PVA increased with increasing time, reaching an equilibrium swelling state in the third hour, which was the starting point of the plateau phase. When the swelling degrees of PVA and AgNPs added to PVA hydrogels were compared, it was observed that increasing the amount of AgNPs in the hydrogels increased the swelling ratio. Swelling is directly proportional to the porosity of the hydrogel networks. The increase in the content of nanoparticles in the hydrogel structure increases the water absorption in the hydrogel structure, leading to an expansion of the hydrogel network and the formation of pores and voids [36,37]. 

#### 2.2.3. Mechanical Test 

To determine the tensile strength of the hydrogels, film samples were cut as rectangular thin films in sizes of 10–30 mm, placed between the jaws of the device, and pulled at a speed of 1 mm/min.

**Figure 9:**
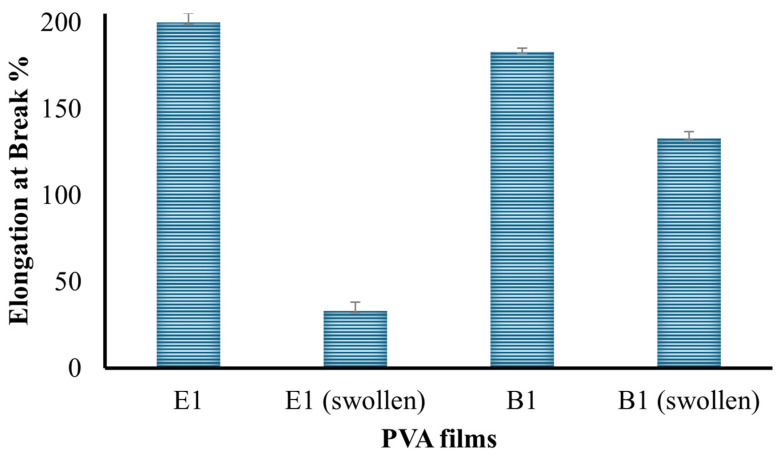
Percent elongation at break of the hydrogels.

**Figure 10:**
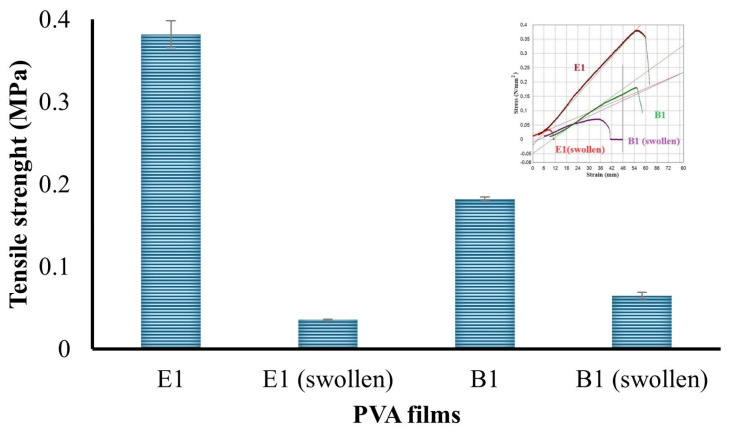
Tensile strength of the hydrogels.

The percent elongation at break of the B1 and E1 films and their swollen forms is given in Figure 9, and the tensile strength (MPa) is given in Figure 10. The PVA hydrogel prepared at a ratio of 10% (*w*/*v*) displayed relatively higher tensile strength than that prepared at 5% (*w*/*v*). The maximum force and elongation at break also exhibited the same behavior, consistent with the literature [38,39]. 

#### 2.2.4. SEM Results

**Figure 11:**
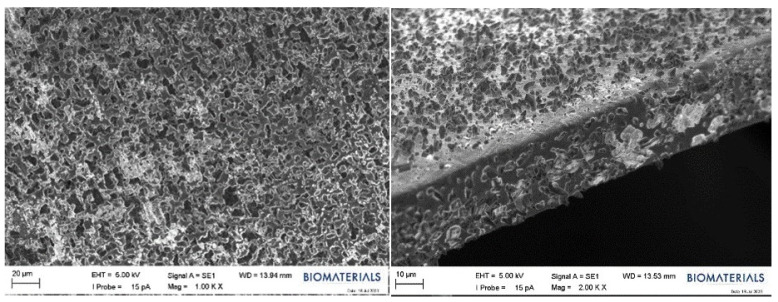
SEM images of the B1 hydrogel film.

**Figure 12:**
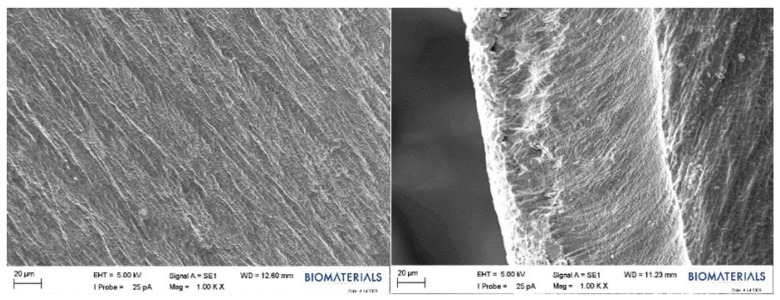
SEM images of the E1 hydrogel film.

Within the scope of this study, SEM analysis of the arranged E1, B1, and swollen E1 hydrogels was performed to obtain information about their morphological structures. Editing of SEM recordings and comparisons of B1 (Figure 11) and E1 (Figure 12) hydrogel films were performed. The E1 hydrogel film exhibited a more homogeneous and integrated structure than the B1 film. The average values decreased as the amount of PVA increased. Owing to the increase in the amount of PVA, the performance of PVA–PVA crosslinks improves when films have a smoother structure [40]. In this study, the freeze–thaw technique was employed three times during the preparation of PVA hydrogels with the aim of achieving the physical self-crosslinking of PVA [41], eliminating the need for a crosslinker. Physical self-crosslinking of PVA without external crosslinking agents has several advantages in certain applications. The process simplifies manufacturing by eliminating the need for additional agents, making it a cost-effective and straightforward option. In addition, the absence of external crosslinkers might be advantageous in applications in which reducing chemical additives is a priority, such as in medical or food-contact scenarios. Self-crosslinked PVA could also exhibit enhanced biocompatibility and reduced toxicity compared to formulations containing external crosslinking agents, making it suitable for specific medical or pharmaceutical applications [42]. It has been observed that the thickness of PVA does not lead to agglomeration of the coating and that the PVA film is distributed homogeneously. A similar result was obtained for different amounts of crosslinked PVA solutions using the freeze–thaw technique [43,44]. The SEM images illustrated that as the PVA ratio increased, the hydrogels became smaller, and their walls became thicker. 

**Figure 13:**
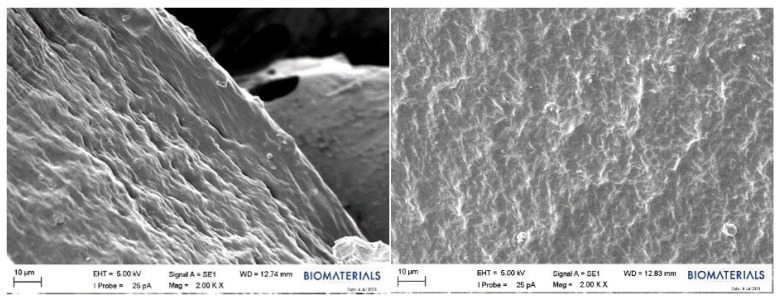
SEM images of the E1 hydrogel in the swollen form.

An SEM image of the swollen state of the E1 hydrogel containing 10% (*w*/*v*) PVA after being kept in PBS is presented in Figure 13. When the SEM images of the E1 film and the swollen E1 film were compared, it was observed that the physical appearance of the E1 film, which has a tight network structure, changed with swelling, and bonding with water molecules was noted. This difference is believed to indicate the presence of swelling and reflect the gel strength of hydrogels [45].

#### 2.2.5. Cumulative Release of Ibuprofen 

**Figure 14:**
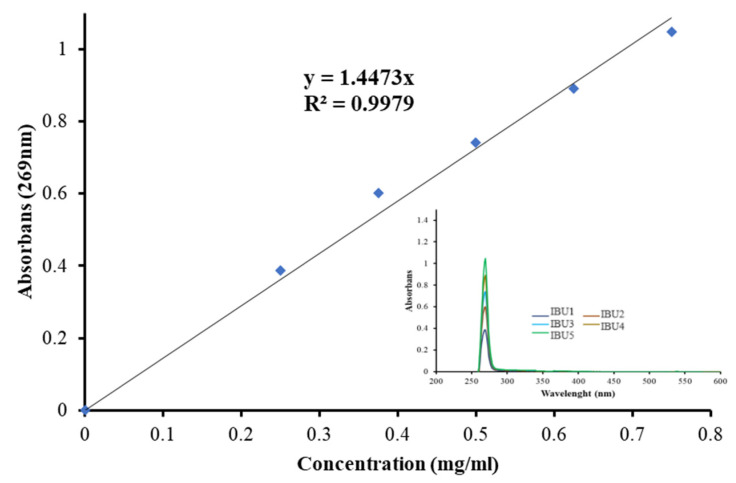
Calibration curve of ibuprofen.

To draw the calibration curve of ibuprofen, samples prepared at concentrations of 0.25, 0.375, 0.5, 0.625, and 0.75 mg/mL were used. The calibration curve and UV–Vis spectra of a series of ibuprofen solutions used to draw the curve are presented in Figure 14. As illustrated in the figure, the characteristic peak of ibuprofen was observed at 269 nm [46]. The calibration curve was drawn using the values obtained at this wavelength in the spectra.

**Figure 15:**
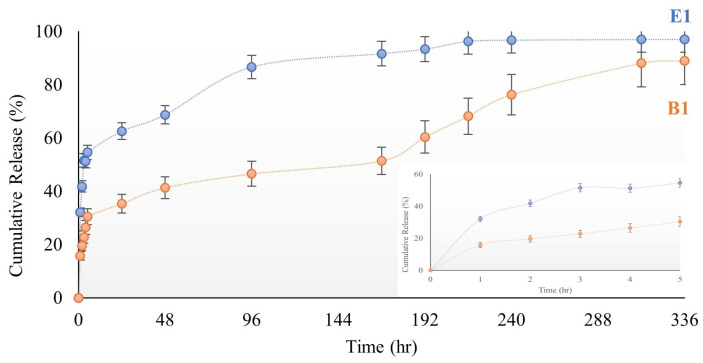
Cumulative release graph of the hydrogel-structured B1 and E1 films.

After the characterization studies, release studies were continued with B1 and E1 hydrogels. The films were loaded with the active ingredient ibuprofen. The amount of drug loaded was calculated by recording the concentration and volume of the remaining drug solution. Based on the swelling test, B1 and E1 films were kept in 2 mL ibuprofen solutions at a concentration of 1 mg/mL for 3 h for drug loading. The drug-loading efficiency of hydrogel films was 65% for the B1 film and 67.5% for the E1 film according to the equation given in the Materials and Methods. Release studies of drug-loaded hydrogels were performed in a shaking incubator at 37 °C and 60 rpm with observation for 336 h. Measurements were taken from drug-loaded hydrogels every hour for the first 5 h. After the first 24 h, measurements were continued every 24 h. This method was applied in drug release experiments using two different films. Cumulative release graphs of B1 and E1 films are presented in Figure 15 [47,48,49].

**Figure 16:**
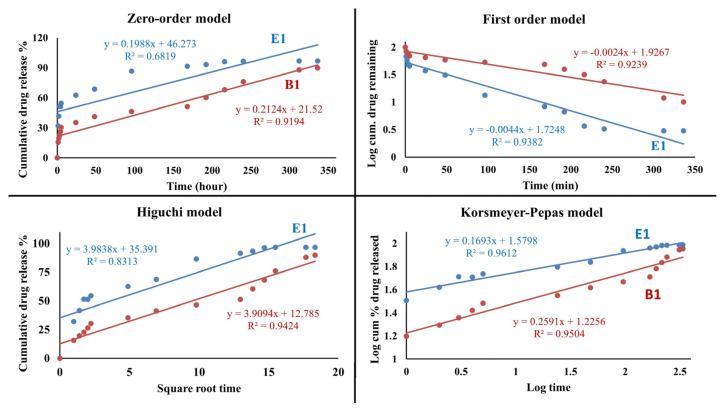
Zero–order, first–order, Higuchi, Korsmeyer–Peppas kinetic models of B1 and E1 films.

As illustrated in the release graphs, increasing the PVA ratio had different effects on the cumulative drug release percentage. After 5 h, the B1 hydrogel film released 30% of the drug, versus 55% for the E1 hydrogel film. The E1 hydrogel film released 97% of the loaded drug on day 10, and the B1 hydrogel film released 89% of the loaded drug on day 14. According to these results, it was understood that the time-dependent drug release increased as the amount of PVA increased. In line with these comparative results, it was believed that if faster release is desired, the E1 film should be utilized, whereas the B1 film should be used if slower release is desired. According to this result, it is thought that the hydrogel-structured film design can be realized within a specified time and according to the desired release amount of the drug. The release profiles of B1 and E1 are fitted to zero-order, first-order, Higuchi, and Korsmeyer-Pepas kinetic models [50,51] to evaluate their release behavior, as shown in Table 1. Based on the R^2^ values, the Korsmeyer-Pepas model represents a more suitable fit. Figure 16 shows the Korsmeyer-Pepas kinetic model for B1 and E1. The adherence to the Korsmeyer -Peppas kinetic model in drug release signifies that the drug release can be expressed by a specific mathematical model. The Korsmeyer -Peppas model is widely employed, particularly in polymer-based drug delivery systems. This model serves to elucidate the release mechanism and how the drug release rate evolves over time.

It can be observed that the release profile of ibuprofen establishes a linear relationship with the square root of time for all samples. Especially prevalent in polymer-controlled drug delivery systems, the Korsmeyer -Peppas model is a valuable tool for understanding the intricacies of drug release mechanisms. Its utility extends to deciphering how the drug release kinetics change with time. A well-fitted Korsmeyer -Peppas model becomes a crucial asset in the design and optimization of drug release strategies, particularly in the realm of pharmaceutical formulations utilizing polymers. In summary, n in the Korsmeyer -Peppas model is 0.1693. It is lower than 0.5 in the Korsmeyer -Peppas model, so it indicates a release mechanism that is highly dominated by Fickian diffusion [53], where the drug release is primarily controlled by the diffusion of the ibuprofen through the polymeric matrix. 

### 2.3. Determination of Cell Viability by In Vitro Cytotoxicity Analysis

**Figure 17:**
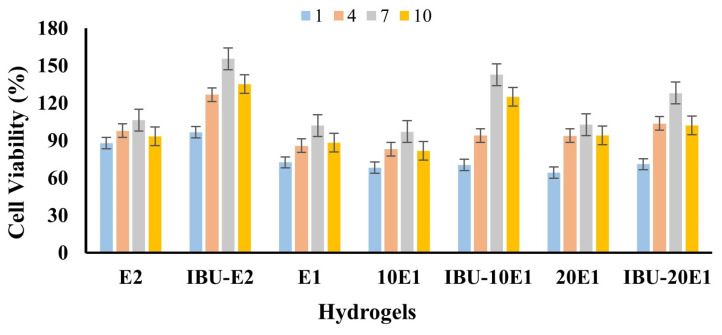
MTT analysis result of samples at the 1st, 4th, 7th, and 10th days.

MTT analysis results of the samples E1 (1 mg AgNPs/PVA), E2 (PVA), IBU-E2 (IBU-PVA), IBU-E1 (IBU-1 mg AgNPs/PVA), 10E1 (10 mg AgNPs/PVA), IBU-10E1 (IBU-10 mg AgNPs/PVA), 20E1 (20 mg AgNPs/PVA), and IBU-20E1 (IBU-20 mg AgNPs/PVA) are given in Figure 17. PVA films included different amount of AgNPs; E1: 1 mg AgNPs, 10E1: 10 mg AgNPs, and 20E1: 20 mg AgNPs. Indirect samples of the prepared films were collected on days 1, 4, 7 and 10 and stored at +4 °C until used. From the MTT analysis results, depending on the days, the cell viability of the E2 film was generally stable, whereas the cell viability of all samples first increased and then decreased over the days. The reason for this finding was that based on the release experiments, the IBU-loaded PVA films released 40–50% of the loaded drug. The cell viability of the E2 film was approximately 90–100%, its biological compatibility was high, in line with the literature, and its activity was stable [54]. 

It was observed that cell viability decreased slightly with the addition of AgNPs into PVA, but cell viability values were generally 64% and above on the first day. All samples containing ibuprofen were both biocompatible and showed increasing viability. When the cytotoxicity of 10E1 and 20E1 films loaded with AgNPs at different rates was evaluated, it was seen that cell viability was lower than the E1 film. Even though it contained 10- and 20- times the amount of silver nanoparticles, cell viability was observed at almost the same level. However, it was evaluated that the films loaded with ibuprofen at all rates showed more viability. The drug loading capacity of the E1 film was determined as 67.5%. During MTT studies, it was determined that 10E1 and 20E1 films had the same loading capacity because the PVA amounts they contained were the same. Further, 1.35 mg of drug was loaded onto film discs, with a diameter of 0.5 cm. In indirect MTT studies, the maximum concentration of ibuprofen in the medium was 0.26 mg/mL (1.27 mM) according to 97% drug release, and 100 µL samples were taken from this medium for MTT analyses. In a study, Ibuprofen kept cell viability at 100% up to 1 mM in KKU-M139 and KKU-213B cells. When the concentration reached 2 mM, cell viability decreased by 50% and 40% in that study [55]. In another study, the impregnation of ibuprofen into the polyhydroxyalkanoate matrix produced by Pseudomonas chlororaphis subs. aurantiaca using supercritical CO_2_ was proposed. The highest ibuprofen content (90.8 ± 6.5 mg ibuprofen/gPHA) was achieved at 20 MPa and 40 °C for 1 h. The release of ibuprofen from the biopolymer was investigated for all impregnation conditions, and 45% of the drug was released within the first hour. Cytotoxicity studies of drug-loaded polymers were conducted on L929 and HaCaT cell lines to confirm the biocompatibility of the biopolymer [56]. According to these studies, the reason why viability did not decrease in our study is that even when the amount of drug released from the films reached 97%, the ibuprofen concentration in the medium was much lower than the value stated in the literature that would reduce cell viability.

### 2.4. Comparison of the Antibacterial Effects of Hydrogel Films 

**Figure 18:**
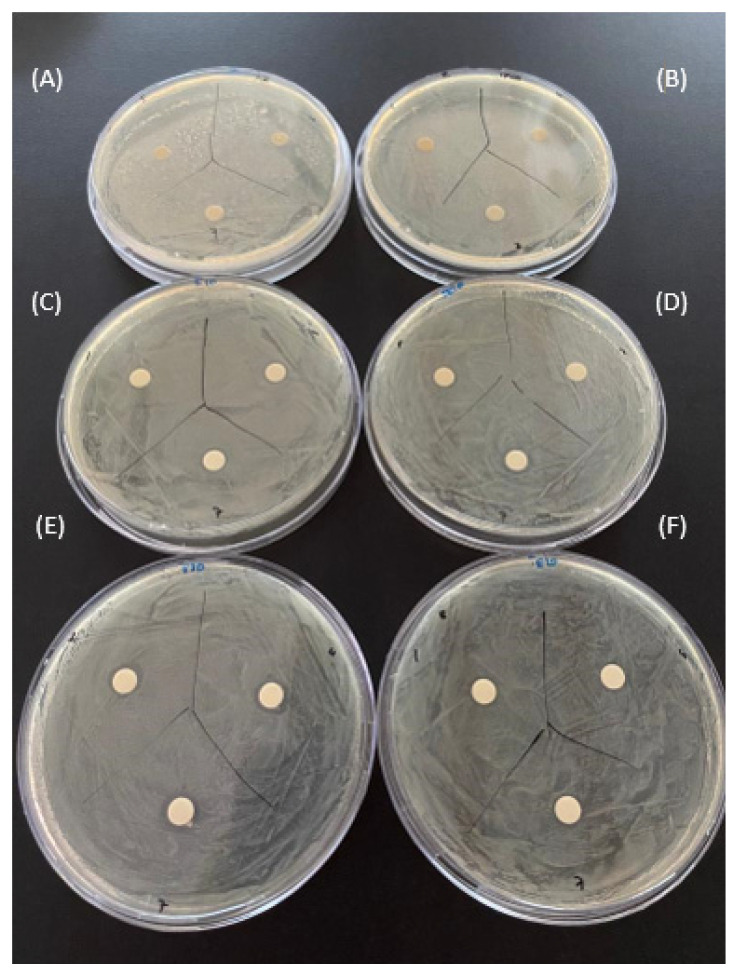
Antibacterial activity test result of hydrogel film sample’s against *E.coli* 1st, 4th, and 7th days. (**A**) E2 (PVA), (**B**) IBU-E2 (IBU-PVA), (**C**) 10E1 (10 mg AgNPs/PVA), (**D**) IBU-10E1 (IBU-10 mg AgNPs/PVA), (**E**) 20E1 (20 mg AgNPs/PVA) and (**F**) IBU-20E1 (IBU-20 mg AgNPs/PVA).

**Figure 19:**
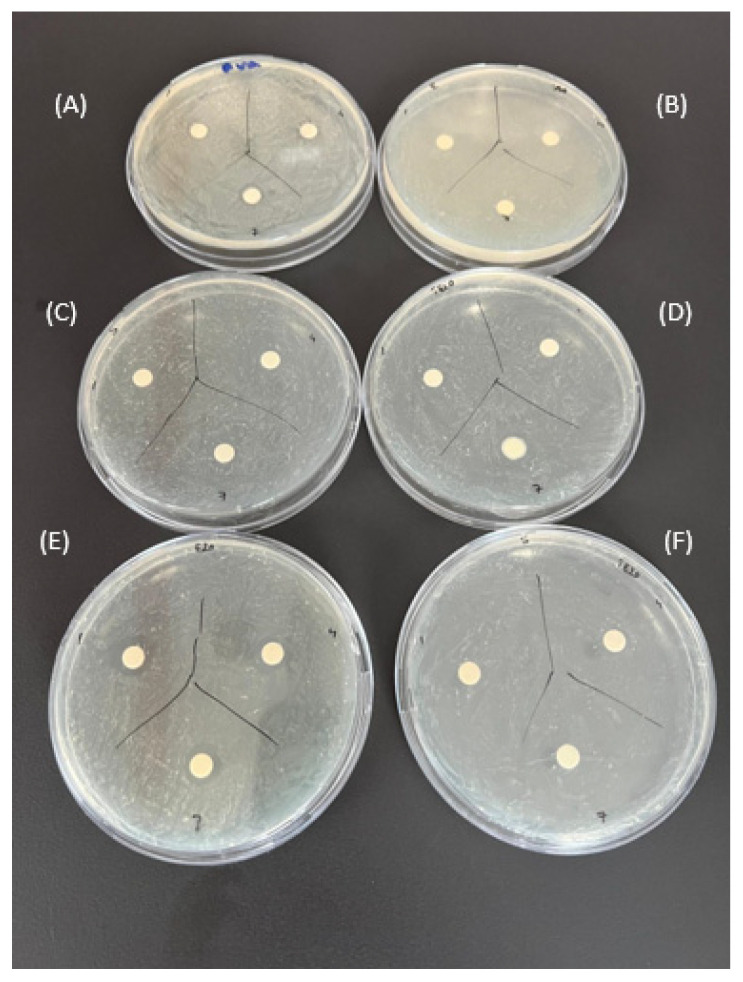
Antibacterial activity test result of hydrogel film sample’s against *S.aureus* 1st, 4th, and 7th days. (**A**) E2 (PVA), (**B**) IBU-E2 (IBU-PVA), (**C**) 10E1 (10 mg AgNPs/PVA), (**D**) IBU-10E1 (IBU-10 mg AgNPs/PVA), (**E**) 20E1 (20 mg AgNPs/PVA) and (**F**) IBU-20E1 (IBU-20 mg AgNPs/PVA).

The in vitro antibacterial activities of the prepared hydrogel film samples used in the in vitro cytotoxicity analysis were examined against Gram-negative *E. coli* and Gram-positive *S. aureus* bacteria. It can be clearly seen from Figure 18 and Figure 19, that PVA hydrogels both not containing IBU (A) and containing IBU (B) did not show a significant antibacterial effect on both bacteria strains. The E1 film containing 1 mg silver nanoparticles did not show an antibacterial effect. For this reason, the amount of silver nanoparticles in the film was increased by 10–20 times and analyses were carried out. In addition, in Figure 18 and Figure 19, no significant antibacterial effect was observed in hydrogel samples not containing IBU (C) and containing IBU (D) with 10 mg of AgNPs. These results are in line with the information regarding the potential antibacterial activity of the produced hydrogels against *E. coli* and *S. aureus* bacteria, in agreement with the findings of K. Swaroop et al. [57]. The antibacterial effect in films with increased silver content is attributed to the well-known antibacterial effects of AgNPs. The antibacterial effects are clearly seen in Figure 18 and Figure 19, including 20 mg of AgNPs in PVA hydrogel film samples (E). In light of the comparative analysis of the antibacterial activity of the films against Gram-positive and Gram-negative bacteria, it is clear that the antibacterial effect against *S. aureus* exceeds the effect against *E. coli.* The evaluation of antibacterial test results shows that increasing the silver content in hydrogel samples has the potential to further enhance the antibacterial effect.

## 3. Conclusions

In conclusion, this study highlights the promising potential of nanoparticle-enhanced polyvinyl alcohol hydrogel systems for controlled drug delivery applications, exemplified by the successful loading and release of ibuprofen from the hydrogel matrices. By utilizing a cost-effective and environmentally friendly approach, films with hydrogel structures were synthesized, exhibiting favorable drug release profiles according to the Korsmeyer–Peppas model. Moreover, the incorporation of silver nanoparticles into these hydrogel matrices demonstrated enhanced antibacterial activity, particularly notable against Gram-positive *S. aureus* bacteria. The observed antibacterial effects underscore the potential of these hydrogel systems in combating bacterial infections, thus expanding their utility in biomedical applications. Future research efforts could focus on optimizing the silver nanoparticle content to further enhance antibacterial efficacy while maintaining biocompatibility. Overall, this study contributes valuable insights to the field of controlled drug release systems and sets a foundation for further advancements in this area of research.

## 4. Materials and Methods

### 4.1. Solution Preparation

#### 4.1.1. Preparation of Ibuprofen Stock Solution 

Ibuprofen (Bilim Pharmaceuticals, İstanbul, Türkiye) was used as the active ingredient in this study. The ibuprofen stock solution was used for subsequent drug loading into the PVA hydrogel films during the experimental procedures. For the primary stock solution, 10 mg of powdered ibuprofen were accurately weighed and dissolved in 10 mL of PBS (Sigma-Aldrich, St. Louis, MI, USA) using a magnetic stirrer for 2.5 h. The pH of the stock solution used in the experiments was measured and recorded as 6.12 [19]. 

#### 4.1.2. Preparation of PVA Solutions 

PVA (molecular weight: 146–186 kDa; 99%) was obtained from Sigma-Aldrich. PVA solutions were prepared at two concentrations, namely 10% (*w/v*) PVA (E2) and 5% (*w/v*) PVA (B2), in PBS using a magnetic stirrer. The solutions were prepared initially without heat for 30 min and then for 90 min at 50–60 °C. To obtain a homogeneous PVA solution, the solution was incubated in an ultrasonic water bath at 40 °C for 90 min. The pH of the solution was 7. 

#### 4.1.3. Extraction of Plant Extract and Green Synthesis of AgNPs

The dried *E. telmateia* plant, obtained from a local herbalist, was ground into a powder using a grinder, resulting in a weight of 20 g. Subsequently, the powdered plant material was combined with 300 mL of distilled water in a beaker and stirred for 4 h on a magnetic stirrer in a temperature range of 70–80 ℃, avoiding boiling. After the steeping process, the mixture was filtered using filter paper, resulting in the extraction of the desired plant extract [58]. 

A solution of silver nitrate (AgNO_3_) was prepared by combining 4 g of AgNO_3_ with 40 mL of distilled water using a magnetic stirrer at room temperature. Concurrently, 250 mL of the plant extract was mixed in a mechanical mixer at 300 rpm at room temperature. The AgNO_3_ solution was then added dropwise to the plant extract using an automatic pipette. The pH of the resulting mixture was adjusted to 11 at room temperature using 1 M NaOH. The resultant mixture was centrifuged at 10,000 rpm for 15 min. Following centrifugation, the precipitate was washed to eliminate liquid plant residues. Specifically, the precipitate was washed once with 97% ethyl alcohol, followed by three washes with distilled water. Subsequently, the residue was dried at 60 °C in a laboratory oven for 48 h to obtain the final product [59]. 

#### 4.1.4. Preparation of AgNP-doped PVA Solutions

In parallel with the films containing only PVA (B2 and E2), PVA films containing AgNPs were also prepared (Table 2). During the preparation of 10% and 5% (*w/v*) PVA solutions, 1 mg of AgNPs was added after 45 min. To ensure that the AgNP-doped PVA solution had a homogeneous structure, it was incubated in an ultrasonic water bath at 40 °C for 90 min. The obtained PVA and AgNP-doped PVA solutions (B1 and E1) were poured into Petri dishes. PVA solutions were dried in an oven at 50 °C for 4 h. PVA solutions taken from the oven were frozen twice at −4 °C for 24 h each [38,60].

### 4.2. Characterization

#### 4.2.1. Particle Size Analysis 

AgNPs were synthesized from *E. telmateia* using the green synthesis method. The particle size (Z-average) of these nanoparticles was determined via dynamic light scattering using a Malvern Zetasizer Nano device.

#### 4.2.2. UV–Vis Absorption Spectroscopy

The spectrum of AgNPs was investigated by scanning across a wavelength range of 100–800 nm at room temperature using a Shimadzu UV mini-1240 spectrophotometer.

#### 4.2.3. FT-IR Spectroscopy

This technique is based on measuring the absorption of electromagnetic radiation by wavenumber in the midinfrared region (4000–400 cm^−1^) [61]. In this study, an FT-IR (IRAffinity-1, Shimadzu) device was used to verify the functional groups of PVA, ibuprofen, and AgNPs.

#### 4.2.4. Swelling Test

The swelling efficiency of the hydrogel is directly related to its microstructure and porosity. For the swelling test, sections were taken from the prepared films, and their dry weights were recorded. Then, each of the cut hydrogel films (circles with a diameter of 0.5 cm) was added separately to 5 mL of PBS buffer solution. Films left to swell were subjected to swelling analysis at room temperature for 48 h. Experiments were performed in 3 repetitions. The weight of the swollen hydrogels was measured after 1, 2, 3, 4, 5, 12, 24, and 48 h, and their masses were recorded. The swelling behavior of hydrogels containing different amounts of PVA was determined, and the swelling ratio equations were calculated using Equation (1) [62] (Ws: weight of swollen hydrogel (mg), Wk: weight of dry hydrogel (mg)).

(1) $$\mathrm{Percent}\;\mathrm{swelling}\;\mathrm{rate}=\frac{\mathrm{Ws}-\mathrm{Wk}}{\mathrm{Wk}}\times 100$$

#### 4.2.5. Mechanical Test

The tensile extension test, which was performed to determine the mechanical properties of the samples, was performed at room temperature using a Shimadzu Universal Testing Device. Film samples were cut as thin rectangular films of 10–30 mm in size and placed between the jaws of the device. The sample’s pulling speed was 1 mm/min. Experiments were performed in 3 repetitions. The tensile–strain curves of the films, elongation amounts at break, and tension values at break were determined [63]. 

#### 4.2.6. SEM Analysis

Thermo Fisher Scientific Quattro S (Microscope version 15.2.2) and Zeiss/Evo 10 (ZEISS SmartSEM) brand SEMs were used to determine the morphology of the AgNPs and prepared PVA hydrogels. To provide electrical conductivity on the surface of the hydrogel films, the samples were first coated in a gold-coating unit, and SEM images were obtained [64].

#### 4.2.7. Loading and Release Studies of Ibuprofen in PVA Hydrogels 

To create the calibration curve for ibuprofen, the UV–Vis spectra of ibuprofen solutions of different concentrations (0.25, 0.375, 0.5, 0.625, and 0.75 mg/mL) were taken using a Shimadzu UV mini 1240 spectrophotometer at a wavelength of 200–800 nm at room temperature. Drug release was measured according to the absorbance at 269 nm [46]. 

Release studies were continued with hydrogels containing 5% and 10% (*w/v*) PVA and AgNPs. The films were loaded with ibuprofen. Experiments were performed in 3 repetitions. By recording the concentration and volume of the remaining drug solution, the amount of loaded drug was calculated using Equation (2) [47].

(2) $$\%\;\mathrm{Drug}\;\mathrm{loading}\;\mathrm{efficiency}=\frac{\mathrm{Total}\;\mathrm{drug}\;\mathrm{amount}\;-\;\mathrm{Remaining}\;\mathrm{drug}\;\mathrm{amount}\;}{\mathrm{Total}\;\mathrm{drug}\;\mathrm{amount}}\times 100$$

The release study was performed by excising 3 × 3 cm^2^ sections from AgNP-doped PVA films loaded with ibuprofen (IBU-AgNP/PVA) followed by incubation in 3 mL of PBS in a shaking incubator at 37 °C and 60 rpm. Then, 2 mL samples were taken from the release medium of the drug-loaded hydrogel and replaced with 2 mL of fresh PBS. The released ibuprofen concentrations were calculated using the calibration curve. The curve of ibuprofen–time graph was fitted to zero–order, first–order, Higuchi, and Korsmeyer–Peppas kinetic models, and the drug release was analyzed.

### 4.3. In Vitro Cytotoxicity Analysis 

L929 mouse fibroblasts were used in the cytotoxicity test. The biocompatibility of PVA films was determined according to the ISO-10993-5 standard [65]. Indirect cell viability analysis was performed using the 3-(4,5-dimethylthiazol-2-yl)-2,5-diphenyltetrazolium bromide (MTT) test to evaluate cell metabolic activity [65]. MTT is actively absorbed into cells and is reduced to colorful, water-insoluble formazan via a mitochondria-dependent reaction. Because of the fragmentation of the tetrazolium ring, the pale yellow MTT dye turns into a dark blue–purple formazan product. The MTT-reducing property of cells is taken as a measure of cell viability. The intensity of the color reaction is correlated with the number of viable cells.

### 4.4. Antibacterial Activity Test 

The activities of the prepared hydrogel films against Gram-negative *Escherichia coli* and Gram-positive *Staphylococcus aureus* were examined [66]. Bacterial colonies were taken from stocks of both species prepared in 5 mL of sterile Mueller–Hinton broth and incubated in a shaking oven at 37 °C for 18 h. After incubation, the bacteria were spread in 100 µL aliquots onto previously prepared Mueller–Hinton agar using a Drigalski spatula. There are various methods to determine the antibacterial activities of the prepared hydrogel films. The agar disc diffusion method was carried out to detect the antibacterial activity [67]. Each set of the PVA films’ 20 µL liquid samples was impregnated onto the antimicrobial susceptibility test discs. The antibacterial activities were observed by incubating the Petri dishes in a 37 °C oven for 24 h. 

## Figures and Tables

**Table 1:** Kinetic models for drug release.

Kinetic Model	*R^2^*		
	Equation	B1	E1
Zero-order	Q_t_ = Q_0_ + K_0_ t	0.9194	0.6819
First order	ln Q_t_ = ln Q_0_ + K_1_ t	0.9239	0.9382
Higuchi	Q_t_ = K_H_ t ^1/2^	0.9424	0.8313
Korsmeyer-pepas	Q_t_ /Q_∞_ = K_k_t^n^	0.9504	0.9612

R^2^: correlation coefficient; Q_t_: amount of drug released in time t; Q_0_: initial amount of drug in the dosage form; Q_∞_: total amount of drug dissolved when the dosage form is exhausted; n: release exponent; K_0_, K_1_, K_k_, K_H_: release rate constants [52].

**Table 2:** Contents of the obtained PVA hydrogel films.

Code	PVA (g)	PBS (mL)	AgNPs (mg)
B1	0.5	10	1
B2	0.5	10	-
E1	1	10	1
E2	1	10	-

## Data Availability

The data supporting the findings of this study are available in the computer systems of Acibadem University for the scanning electron microscopes, UV-Vis spectrophotometer, and mechanical test analyses, in the computer systems of Yeni Yüzyıl University for the UV-Vis and FT-IR spectrophotometer analyses, and in the computer systems of Yildiz Technical University Bioengineering Department for the Zeta analyses. Access to these data may be granted upon request and in accordance with the respective data access policies of each institution.
